# Maintenance of prehospital anaesthesia using an intermittent bolus regime in blunt trauma patients with a high GCS and hemodynamic reserve: a retrospective cohort study

**DOI:** 10.1186/s13049-026-01654-w

**Published:** 2026-06-25

**Authors:** Brad Sheridan, Joanne Griggs, Pedro Mondim, Ewoud ter Avest, Richard Lyon

**Affiliations:** 1 John Hunter Hospital and Hunter Retrieval Service, Newcastle, Australia; 2 Injury and Trauma Research Program, Hunter Medical Research Institute, Newcastle, Australia; 3 Air Ambulance Charity Kent Surrey Sussex, Redhill Aerodrome, Redhill, UK; 4 University of Surrey, Duke of Kent Building, Guildford, UK; 5 Department of Acute Care, University Medical Center Groningen, Groningen, The Netherlands; 6 London’s Air Ambulance, London, UK

**Keywords:** Anaesthesia, Emergency medical services, Fentanyl, Ketamine, Midazolam, Prehospital, Rocuronium, Sedation

## Abstract

**Background:**

In-hospital intravenous maintenance of anaesthesia is commonly achieved by continuous infusion, while prehospital maintenance of anaesthesia after rapid sequence induction (RSI) is currently commonly provided by intermittent boluses. However, the practice effect of this approach has never been fully established.

**Methods:**

In a single centre retrospective observational study at a helicopter emergency medical service (HEMS) in the UK, post-RSI anaesthesia maintenance by intermittent boluses was studied between 1 January 2019 and 28 February 2023. Time- and weight-adjusted average maintenance drug doses (as a surrogate for efficacy of sedation) were calculated for a consecutive sample of 101 blunt trauma patients receiving a prehospital anaesthetic with a Glasgow Coma Scale (GCS) ≥ 9 and without significant cardiovascular instability. Associations between (cumulative) maintenance drug doses and induction doses, patient characteristics, physiological parameters, and anaesthesia duration were evaluated using Pearson correlation coefficients and 95% confidence intervals.

**Results:**

Median time of prehospital anaesthetic duration was 56 min (range 16–116 min). Maintenance dosing of fentanyl clustered around a median [IQR] dose of 0.64 [0-1.41] mcg/kg/h (minimum non-zero: 0.26mcg/kg/h, maximum: 5.56 mcg/kg/h). For midazolam, this was 0.028 [0-0.041] mg/kg/h (minimum non-zero: 0.005 mg/kg/h, maximum 0.157 mg/kg/h), and for ketamine 0 [0-0.23] mg/kg/h (minimum non-zero 0.05 mg/kg/h, maximum: 1.62 mg/kg/h). Eight patients (duration of anaesthesia between 48 and 74 min), received no maintenance sedation or analgesic agents. No clinically relevant associations were identified between maintenance dosing of any agent and induction dosing, patient demographics or pre-induction physiological parameters.

**Conclusion:**

This retrospective analysis of a homogeneous cohort of patients with haemodynamic reserve and a GCS ≥ 9 undergoing prehospital emergency anaesthesia showed that anaesthesia maintenance by an intermittent bolus-only regimen comes with notable variability in cumulative doses administered and may result in a potential risk of sub-therapeutic plasma levels.

**Supplementary Information:**

The online version contains supplementary material available at 10.1186/s13049-026-01654-w.

## Background

Prehospital emergency anaesthesia (PHEA) is a critical intervention for patients with significant traumatic injuries and has been shown as a safe alternative to in-hospital anaesthesia [1, 2]. Further, PHEA may contribute to improved outcomes in patients with severe traumatic brain injury (TBI) [3], by facilitating the early institution of important interventions such as securing a definitive airway and controlling oxygenation and ventilation [1, 3–5].

While there are numerous recommendations on the process of PHEA induction [6–8] and on post-induction management of ventilation and haemodynamics [5, 7, 9], there is limited consensus in the literature on the subsequent maintenance of anaesthesia in the prehospital setting [10]. Maintaining adequate levels of anaesthesia and avoiding under-sedation post-PHEA may incur numerous benefits to the prehospital trauma patient, including reducing intracranial pressure and decreasing cerebral metabolic demand [11]; attenuating the hyper-adrenergic response to trauma and reducing subsequent sequalae such as multiple organ dysfunction syndrome (MODS) [12–14]; and avoiding awareness and consequent psychological trauma [15–17].

While in-hospital intravenous maintenance of anaesthesia is achieved by continuous infusion, in the prehospital setting, anaesthesia maintenance by an intermittent bolus regime remains common practice [7, 8, 10, 18]. However, bolus administration of sedatives carries the potential risks of undersedation and awareness, as dosing may invertedly be delayed or entirely omitted [7, 10, 14, 18–20]. Most services utilising intermittent boluses do not provide recommendations on maintenance bolus intervals and/or dosing [10], which may contribute to significant inter-clinician variability and potential underdosing [10, 14].

In the present study, we use total administered drug dosing standardised to weight and time (i.e. m(c)g/kg/hr) to study the practice effect of an intermittent bolus regime on maintenance of prehospital anaesthesia in trauma patients who, based on their clinical characteristics, would be expected to require a reasonable level of post-RSI anaesthesia.

## Methods

### Study design and population

A retrospective observational study was performed of prehospital trauma patients who received PHEA by a single UK HEMS service (Air Ambulance Kent Surrey Sussex (KSS)) and were transferred to either a Major Trauma Centre (MTC) or Trauma Unit (TU) between 1 January 2019, and 28 February 2023. To investigate practice (rather than clinical) variation, a homogenous cohort of haemodynamically stable blunt trauma patients without severe traumatic brain injury (TBI) were selected. Eligible patients were ≥ 12 years of age and underwent PHEA with a pre-induction GCS of 9–15 (as these patients are expected to require a reasonable dose of induction and maintenance drugs). Patients were excluded if their pre-induction systolic blood pressure (SBP) was < 100mmHg, or pre-induction shock index (SI) was > 1.0 (to exclude cerebral hypoperfusion as a significant contributor to any reduced level of consciousness, and to ensure patients had enough haemodynamic reserve to allow adequate doses of induction and maintenance drugs). Patients who received excessive doses of ketamine or fentanyl (greater than 150% of the maximum recommended induction dosing per KSS PHEA Standard Operating Procedure (SOP)) prior to HEMS arrival were excluded, as this would confound subsequent dosing and was outside control of the HEMS team. There were no deaths during our care in this patient cohort.

### Study setting

KSS covers a region of three counties in the southeast of England with a resident population of 4.5 million and transient population of up to 8 million. Two prehospital emergency medicine teams respond 24 h a day, 7 days a week by either helicopter or rapid response vehicle. Physicians have a minimum of 5-years postgraduate experience, including a minimum of 6-months hospital anaesthesia training. Paramedics undergo critical-care paramedic training, including theoretical modules on RSI. Prior to independent prehospital practice, medical crew undergo an intense training period including structured medical education, training, and operational supervision by prehospital care consultants. During this period, training is focused to ensure crews are competent at performing safe PHEA. The HEMS team adheres to SOPs, which govern all aspects of prehospital practice, including PHEA.

### Clinical practice during the study period

Consistent with peer-reviewed literature [1, 21–23], there are 6 indications for PHEA outlined in the KSS SOP, namely: (1) actual or impending airway compromise; (2) ventilatory failure; (3) unconsciousness; (4) patients who are unmanageable or severely agitated after head injury; (5) anticipated clinical course, whereby a patient is likely to deteriorate on route to hospital, and; (6) humanitarian need. The process of PHEA induction as per the KSS SOP has been described in detail elsewhere [6, 22]. Since 2012, the KSS PHEA SOP has stipulated a standardised ratio-based RSI dose regimen [6, 22], and in February 2020 the rocuronium induction dosing increased from 1 mg/kg to 2 mg/kg [24, 25]. However, during the study period there was no detailed institutional guidance for the provision of anaesthesia maintenance. Clinical practice was to provide this anaesthetic maintenance using intermittent boluses, and the drug choice, dose, and timing of these were at the discretion of each attending clinician. Further, the study period covers a time when both non-invasive arterial blood pressure (NIBP) and invasive arterial blood pressure (IABP) were used to monitor patient haemodynamics, with IABP being introduced for suspected TBI patients at the discretion of the clinician from January 2022 [26].

### Data acquisition and definitions

The electronic medical records of patients meeting the inclusion criteria were retrospectively interrogated from the KSS Electronic Patient Clinical Record (ePCR) HEMSbase [Version 2.0, MedicOne]. Data collected included: basic demographics [age, estimated weight, sex]; mechanism of injury; pre-induction clinical observations [HR, SBP, GCS]; dose [mg or mcg] and type of pre-induction sedatives and opioids; dose and type of induction sedatives, opioids, and neuromuscular blocking drugs (NMBDs); and dose and type of maintenance sedatives, opioids, and NMBDs.

For each patient the duration of anaesthesia was established by subtracting the time of PHEA from the time of removal of monitoring after handover in-hospital, as recorded in the ePCR. Shock index was calculated by dividing pre-induction HR by pre-induction SBP. Induction doses for each drug were calculated by summing the pre-induction and induction doses, as KSS PHEA practice during the study period was to subtract pre-induction analgesia/sedation from intended induction dosing. Summated induction doses were divided by estimated total body weight to calculate the induction dose per kilogram (dose/kg). Similarly, anaesthetic maintenance dosing was calculated by summing post-RSI bolus doses of a drug and dividing the total by the estimated total body weight and by duration of anaesthesia, to give a dose per kilogram per hour equivalence (dose/kg/h).

### Primary and secondary objectives

The primary objective was to describe the practice effect of an intermittent bolus dosing regimen using midazolam, ketamine, fentanyl and rocuronium for maintenance of anaesthesia post-RSI by establishing (i) the two most common (absolute) bolus doses administered and (ii) total dose, standardised to weight and time (i.e. m(c)g/kg/h), administered.

The secondary objective was to explore the relation between this standardised maintenance dosing and the induction dosing, patient demographics, hemodynamic characteristics, and duration of prehospital anaesthesia.

### Statistical analysis

Descriptive statistics are given as median [IQR] and frequencies with percentages. Statistical correlations were explored between summative induction drug doses in m(c)g/kg/h, SI, HR, GCS, estimated weight, and age and calculated maintenance drug doses in m(c)g/kg/h using Pearson correlation coefficients with significance testing. Data were pre-processed in Microsoft Excel (Microsoft Corp, Redmond, WA USA). Statistical analysis and visualisations were performed using R version 4.3 (R Core Team, 2023. R: A language and Environment for Statistical Computing. R Foundation for Statistical Computing, Vienna, Austria). Statistical significance was pre-determined at *p* < 0.05, and Bonferroni corrections were applied for multiple comparisons.

### Ethical considerations

This project was registered with the University of Surrey and met National Institute for Healthcare Research (NIHR, UK) criteria as a service evaluation. All the data used for this study were routinely collected as part of standard prehospital patient data collection. The project was approved by the KSS Research and Innovation Committee.

## Results

### Characteristics of the study population

**Fig. 1:**
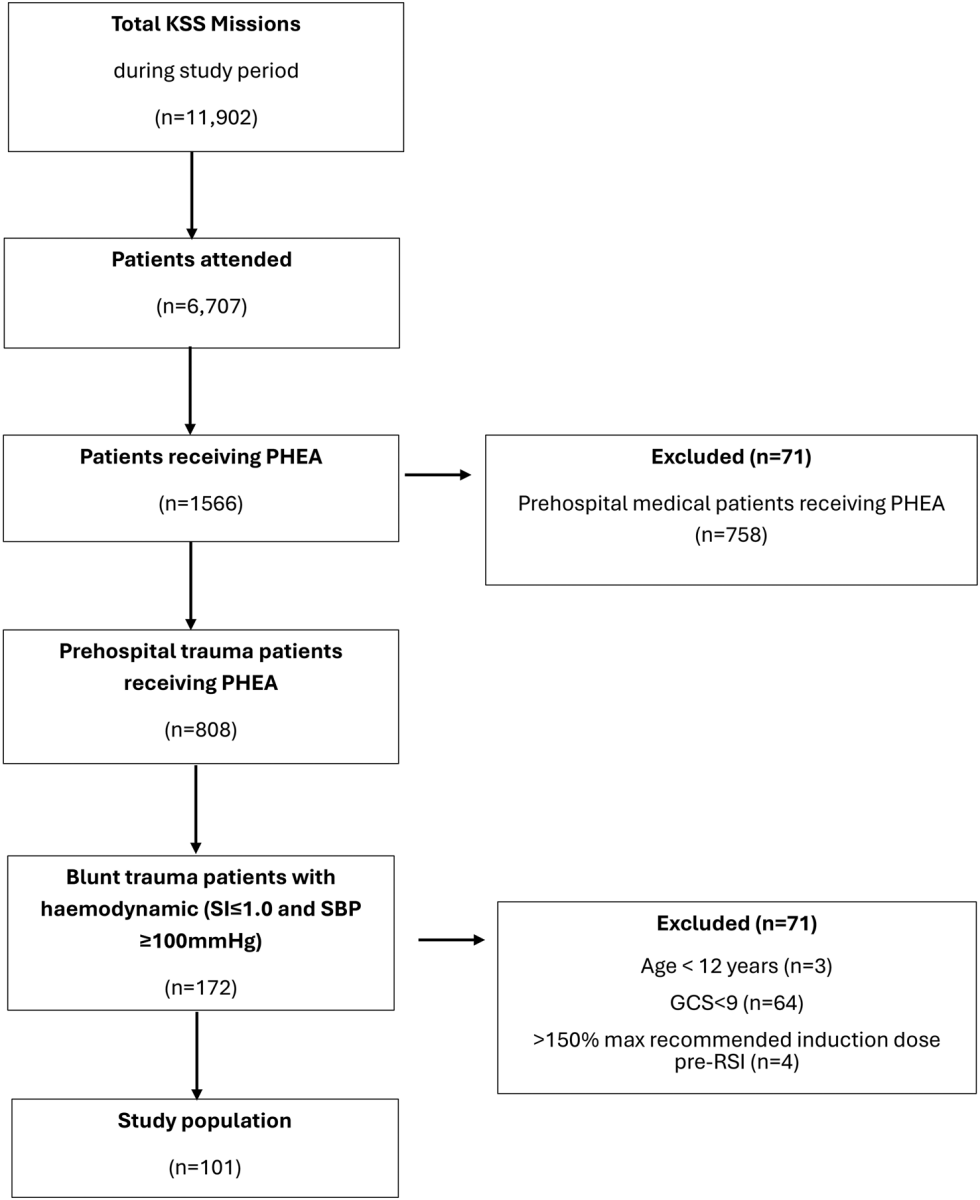
CONSORT diagram. KSS, Air Ambulance Charity Kent Surrey Sussex; SI, shock index; SBP, systolic blood pressure; RSI, rapid sequence intubation; GCS, Glasgow Coma Score

During the study period KSS attended 6707 patients. A total of 101 patients met inclusion criteria (Fig. 1). The majority of included patients were male, with a median age of 52 years [IQR 30–64]. The most common mechanism of injury was road traffic collision (*n* = 40, 39%). Pre-induction vital signs of the study population are represented in Table 1.

**Table 1:** Demographic and clinical characteristics of the study population (*n* = 101)

Characteristic	Study population (*n* = 101)
Male, number (%)	76 (75.2)
Age (years), median (IQR)	52 (30–64)
Weight (kg), median (IQR)	80 (70–90)
GCS, median (IQR)	11 (9–13)
Pre-RSI SBP (mmHg), median (IQR)	142 (123–161)
Pre-RSI HR (bpm), median (IQR)	92 (79–115)
SI, median (IQR)	0.7 (0.5–0.8)

IQR, Interquartile range; GCS, Glasgow Coma Score; RSI, Rapid Sequence Induction; SBP, systolic blood pressure; HR, heart rate; SI, shock index

### Induction, and maintenance of anaesthesia

The median duration of prehospital anaesthesia was 56 min (range 16–116 min). In all patients, induction of anaesthesia was performed with ketamine (*n* = 101) and rocuronium (*n* = 101), with or without fentanyl (*n* = 93). The allocation and co-administration patterns of maintenance anaesthetic agents are shown in Table 2, where fentanyl (*n* = 65 patients, 64.4%) and midazolam (*n* = 61, 60.4%) were the most common maintenance agents, and the most common maintenance combination (*n* = 43, 42.6%).

For maintenance with fentanyl (total of *n* = 65 doses), the most prevalent doses administered were 100mcg (*n* = 12, 18.5%) and 50mcg (*n* = 8, 12.3%). For maintenance with ketamine (*n* = 29), these were 20mg (*n* = 7, 24.1%) and 30mg (*n* = 3, 10.3%), and for maintenance with midazolam (*n* = 61) these were 2mg (*n* = 18, 29.5%) and 1mg (*n* = 11, 18.0%). For maintenance of paralysis, the two most frequent rocuronium doses were 40mg (*n* = 21, 30.0%) and 30mg (*n* = 12, 17.1%).

**Fig. 2:**
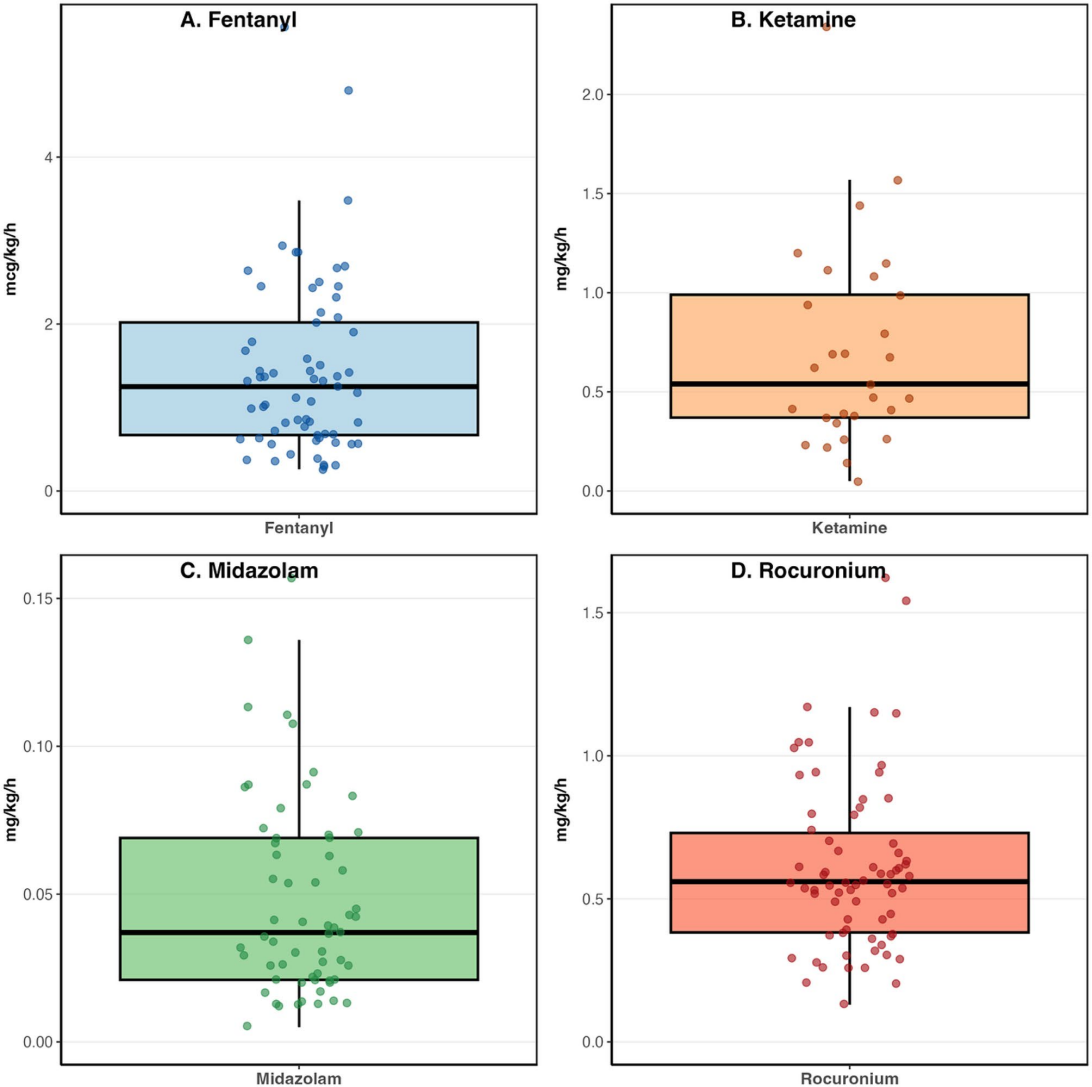
Maintenance dose variation of fentanyl, midazolam, ketamine and rocuronium in trauma patients with a GCS ≥ 9, pre-induction systolic blood pressure (SBP) ≥100mmHg, and pre-induction shock index (SI) ≤ 1.0. (*n* = 101). (**A**) Fentanyl maintenance dosing, (**B**) Ketamine maintenance dosing, (**C**) Midazolam maintenance dosing, (**D**) Rocuronium maintenance dosing. Box plots and jitter showing median (line), interquartile range (box), and individual patient values (dots) for (**A**) fentanyl, (**B**) ketamine, (**C**) midazolam, and (**D**) rocuronium maintenance dosing

The median, minimum non-zero, and maximum doses of pre-induction, induction, and maintenance doses of sedative, analgesic and paralytic agents are shown in Table 3. Box plots with jitter demonstrating the distribution of average standardised maintenance doses of fentanyl, ketamine, midazolam and rocuronium are represented in Fig. 2. These demonstrate a considerable degree of variation in maintenance dosing patterns of all four agents, with fentanyl having the largest spread between minimum non-zero and maximum maintenance dosing (0.26mcg/kg/h to 5.56 mcg/kg/h).

Of note, eight (8%) patients, with a length of anaesthesia between 48 and 74 min, received no maintenance hypnotic or opioid agents. Four of these patients received maintenance paralysis. In two of these eight cases there were documented episodes of post-PHEA hypotension, however in the remaining six patients there was no documented or inferable reason for withholding maintenance sedation or analgesia. A further eight (8%) patients, with a length of anaesthesia between 26 and 66 min, received fentanyl-only or fentanyl- and rocuronium-only maintenance, and hence received no further hypnotic agent post-induction (Table 2).

**Table 2:** Allocation and co-administration of maintenance anaesthetic agents

Treatment Category	Maintenance Agent/Combination	*n*	%	Median Duration
Individual Agents	Fentanyl	65	64.4	56
	Ketamine	29	28.7	57
	Midazolam	61	60.4	57
	Rocuronium	70	69.3	57
Sedation/Analgesia Combinations	Fentanyl ± Rocuronium only	8	10.9	57
	Ketamine only	14	13.9	52
	Midazolam only	10	9.9	61
	Fentanyl + Ketamine	7	6.9	67
	Fentanyl + Midazolam	43	42.6	56
	Ketamine + Midazolam	4	4.0	80
	Fentanyl + Ketamine + Midazolam	4	4.0	68
	No sedation/analgesia	8	7.9	59
Paralysis and sedation patterns	Rocuronium + sedation/analgesia	67	66.3	57
	Rocuronium alone (no sedation/analgesia)	4	3.0	51
	No rocuronium maintenance	31	30.7	56

Frequency (*n* and percentage) and median anaesthesia duration for patients receiving individual maintenance agents and their co-administration patterns. This table describes treatment allocation decisions (which patients received which drugs and in what combinations) and is presented separately from dosing statistics (Table 3) to clearly distinguish between treatment administration and dosing intensity. Eight patients received no maintenance sedation or analgesia; four of these also received rocuronium for paralysis maintenance only

**Table 3:** Pre-induction, induction, and maintenance doses of sedative, analgesic and anaesthetic agents (non-zero doses only)

	Fentanyl	Ketamine	Midazolam	Rocuronium
**Pre-induction**	**(mcg/kg)**	**(mg/kg)**	**(mg/kg)**	**(mg/kg)**
Median (IQR)	0 (0)	0.29 (0–1.00)	0.012 (0-0.017)	0 (0)
Minimum non-zero	0.31	0.13	0.006	0
Maximum	0.83	2.89	0.143	0
Coefficient of Variation	0.37	0.67	0.87	0
**Induction**	**(mcg/kg)**	**(mg/kg)**	**(mg/kg)**	**(mg/kg)**
Median (IQR)	2.86 (1.00–3.00)	1.5 (1.00–2.00)	0 (0)	2 (1–2)
Minimum non-zero	0.83	0.5	0	0.91
Maximum	3.5	2	0	3
Coefficient of Variation	0.34	0.31	0	0.27
**Maintenance**	**(mcg/kg/h)**	**(mg/kg/h)**	**(mg/kg/h)**	**(mg/kg/h)**
Median (IQR)	0.64 (0-1.41)	0 (0-0.23)	0.028 (0-0.041)	0.43 (0-0.63)
Minimum non-zero	0.26	0.14	0.012	0.13
Maximum	5.56	2.34	0.157	1.62
Coefficient of Variation	0.73	0.73	0.70	0.49

Median [interquartile range], minimum non-zero, and maximum pre-induction, induction, and maintenance doses of fentanyl, ketamine, midazolam, and rocuronium. Statistics shown only for patients who received each respective agent (non-zero doses). IQR, interquartile range. Coefficient of variation (CV = standard deviation/mean) shown for non-zero doses to indicate relative dosing variability

### Maintenance dosing in relation to relevant patient characteristics, induction doses, and time-to definitive care

**Fig. 3:**
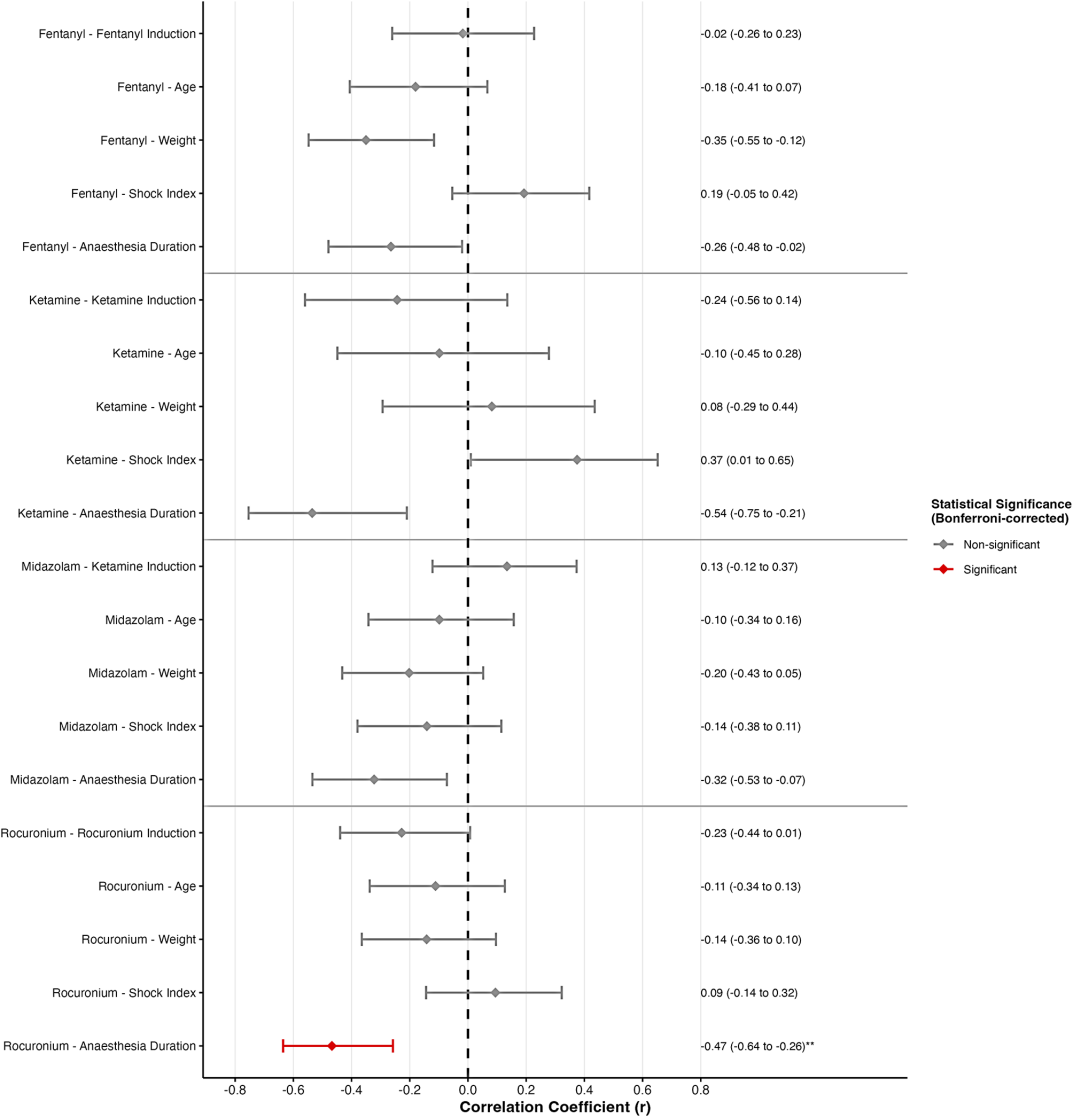
Correlations between maintenance doses and induction doses, patient demographics, shock index, and anaesthesia duration. Forest plot showing Pearson correlation coefficients (r) with 95% confidence intervals for associations between standardised per-kilogram-per-hour maintenance doses of fentanyl, ketamine, midazolam, and rocuronium with induction dosing, age, weight, shock index, and anaesthesia duration (complete maintenance dosing-induction dosing associations and maintenance dosing-physiological parameter associations are provided in Supplementary Tables S1 and S2, respectively). Significant correlations (*p* < 0.05) shown in red; non-significant correlations shown in grey. The dashed vertical line represents no correlation (*r* = 0). Asterisks denote statistical significance: *p < 0.05, **p < 0.01, ***p < 0.001. Only patients receiving each respective maintenance drug are included in correlations (fentanyl *n* = 65, ketamine *n* = 29, midazolam *n* = 61, rocuronium *n* = 70)

Correlations between time- and weight-standardised doses of maintenance drugs and relevant induction doses and patient characteristics are depicted in Fig. 3. After Bonferroni correction for multiple comparisons (20 tests), maintenance dosing showed no significant associations with patient age, weight, haemodynamic status (shock index), or induction dosing. Correlations between induction doses of each drug and maintenance doses of each drug are depicted in Supplementary Table S1, where no significant associations were found. Similarly, no associations were observed between maintenance dosing and pre-induction GCS, pre-induction heart rate, or pre-induction systolic blood pressure (Supplementary Table S2).

**Fig. 4:**
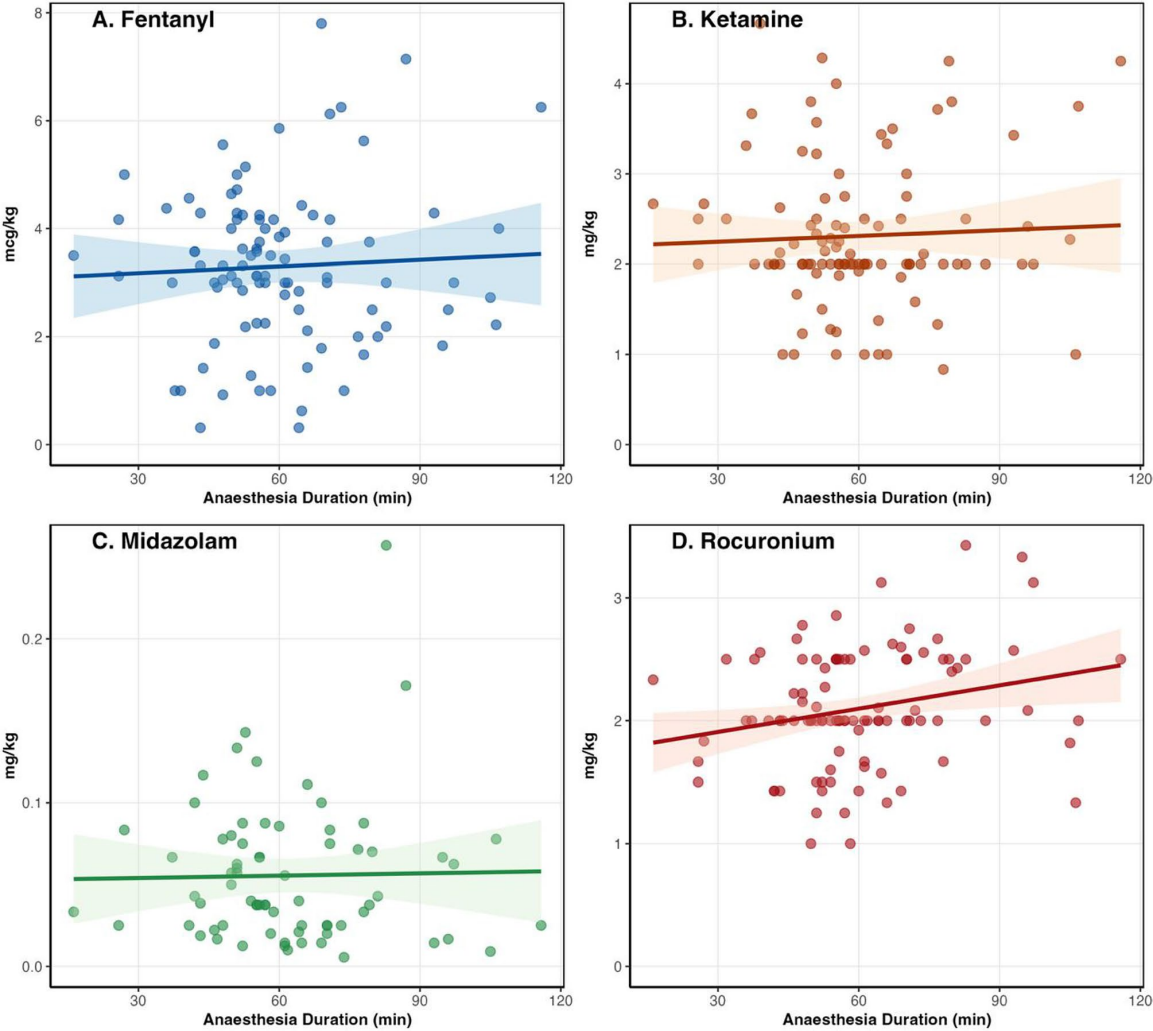
Associations between maintenance dosing and anaesthesia duration. Scatter plots showing the relationship between total cumulative per-kilogram maintenance doses and pre-hospital anaesthesia duration for (**A**) fentanyl (*n* = 65), (**B**) ketamine (*n* = 29), (**C**) midazolam (*n* = 61), and (**D**) rocuronium (*n* = 70). Linear regression lines with 95% confidence intervals demonstrate no significant associations between total cumulative doses and duration for any drug. This analysis using total dose (mg/kg) addresses the mathematical coupling present in time-normalised dose analyses, demonstrating that clinicians administer consistent total drug quantities independent of transport duration

Total cumulative maintenance drug doses were examined in relation to anaesthesia duration to address potential mathematical coupling inherent in time-normalised dose metrics. Linear regression analysis revealed no significant associations between total cumulative doses (mg/kg) and duration of pre-hospital anaesthesia for fentanyl (*r* = -0.04, *p* = 0.68), ketamine (*r* = 0.07, *p* = 0.71), midazolam (*r* = 0.01, *p* = 0.95), or rocuronium (*r* = 0.15, *p* = 0.19). These relationships are illustrated in Fig. 4, which demonstrates that clinicians administered consistent total drug quantities independent of transport duration.

## Discussion

Our retrospective analysis of prehospital trauma patients with GCS ≥ 9 and haemodynamic reserve undergoing PHEA showed considerable variability in the provision of anaesthesia maintenance when using an intermittent bolus regime. This variability was not attributable to clinical patient characteristics or induction dosing. Notably, total cumulative maintenance drug doses were not significantly related to anaesthesia duration, indicating that clinicians administered similar total drug quantities regardless of transport time. Maintenance doses were generally lower than what would be expected to achieve adequate levels of anaesthesia in haemodynamically stable trauma patients without severe TBI.

Maintaining adequate anaesthesia for patients during their prehospital care is important to reduce cerebral metabolic demand and avoid spikes in intracranial pressure [11], dampen the hyper-adrenergic response to the initial traumatic injury and subsequent noxious stimuli [12–14], and avoid awareness and post-traumatic stress [15–17]. To achieve this in a patient population without severe TBI or significant pre-induction haemodynamic instability, reasonable doses of induction and maintenance anaesthesia would be expected.

In our cohort, the average induction dose of ketamine was 1.5 mg/kg and median duration of anaesthesia was 56 min. Given the plasma concentration of ketamine will drop below a therapeutic sedative threshold after 30 min following a 2 mg/kg induction dose [10], most patients in our cohort would likely require ongoing prehospital anaesthesia maintenance. While most patients received some form of maintenance anaesthesia, based on previously published studies and guidelines, the median (standardised to weight and time) and absolute maintenance doses of midazolam and fentanyl were lower than expected [8, 14, 16, 27] [Appendix 1]. For example, in a mixed medical and trauma population attended by a private South African prehospital service, de Kock et al. described post-intubation maintenance regimens of single-agent midazolam boluses between 3.6 mg to 4.4 mg, and combined-agent midazolam/morphine boluses of 3.2/3.0 mg to 3.6/3.6 mg, with total median times between maintenance boluses of between 10 and 15 min [14]. Further, in the 2016 guidelines of the Out-of-Hospital Working Group for the German Society of Anaesthesiology and Intensive Care, Hossfeld et al. recommended midazolam boluses of 3 to 5 mg and fentanyl boluses of 150mcg repeated every 20 min for anaesthesia maintenance in post-PHEA trauma patients [8]. Of note, while a 2022 practice survey of HEMS services in Australia, New Zealand and the United Kingdom showed intermittent boluses of midazolam between 1 and 3 mg and of fentanyl between 10-40mcg were recommended for trauma patients post-PHEA, there was limited guidance on dosing intervals [10]. Based on our findings, the average patient (with body weight of 80 kg) would receive only 2.2 mg of midazolam and 51.2mcg of fentanyl in one hour. Although the effects of dose-timing and synergism of different agents cannot be evaluated, as exact administration times are not recorded, these doses may have resulted in sub-therapeutic plasma concentrations and inadequate anaesthesia levels for this cohort of patients [10, 14, 16, 19].

A second important finding is the considerable variation in dosing for maintenance of anaesthesia post-RSI. The lack of relationship between total cumulative dose and anaesthesia duration suggests that dose variation is not explained by clinicians titrating maintenance based on transport time. As our data does not have adequate detail to examine the timing of each intermittent maintenance bolus, to assess the temporal association between a bolus and the patient’s physiology, and as it does not allow us to account for potential synergistic effects of multiple agents given over the duration of care, a defendable explanation for the high degree of dose-variation is that clinicians were employing precision-based medicine to optimise the individual patient’s care. However, this argument of precision-based medicine is less compelling when considering the variability in rocuronium dosing. While the change from 1 mg/kg to 2 mg/kg for rocuronium induction dosing during this study period may account for some variation, a doubling of the induction dose cannot solely account for the high degree of variation in maintenance dosing (minimum non-zero dosing 0.13 mg/kg/h vs. maximum 1.62 mg/kg/h, coefficient of variance 49%). Rocuronium dosing should be dependent on the patient’s weight and duration of anaesthesia and not significantly influenced by patient haemodynamics or level of consciousness.

Several factors may contribute to the relative under-dosing and considerable variation observed in anaesthesia maintenance for our cohort, including haemodynamic concerns and clinician workload. The provision of anaesthesia maintenance is always undertaken within the context of competing pharmacodynamic goals. In trauma, an emphasis on achieving haemodynamic goals, such as the avoidance of haemodynamic collapse in the exsanguinating patient [21, 28], and the avoidance of undue hypotension in severe TBI [5], often competes with and can outweigh the provision of adequate anaesthetic depth [14, 20, 28]. However, not all trauma patients undergoing PHEA are exsanguinating or have severe TBI, and in such cases, we might expect the provision of adequate anaesthetic depth to be elevated within the hierarchy of pharmacodynamic considerations. Further, the logistical limitations, and cognitive and technical demands of prehospital practice produce an environment where clinicians are susceptible to cognitive overload [29]. Eight patients received no post-RSI maintenance sedatives or opioids, six of which had no discernible physiological explanation for this omission, and a further eight patients received fentanyl without a further hypotonic agent post-induction. As the sedative and analgesic agents used are controlled drugs, it is highly unlikely that undocumented boluses were given to these patients.

A potential solution that can ensure the continuous provision of adequate levels of anaesthesia, reduce variations in both dosing and pharmacodynamic effects, and offload cognitive burden, is to provide maintenance of anaesthesia post-RSI by continuous infusion, rather than by repeated bolus administration [10]. While a number of services have adopted this practice, there is no consensus on appropriate drug choice or dose, nor efficacy or safety data, hence warranting further research into this area of prehospital care [10].

There are several limitations to this retrospective observational analysis. First, our service operates with teams of two clinicians (paramedic and doctor) who deal with critically ill patients, making live documentation challenging when multiple clinical actions are being carried on concurrently. Hence, as discussed, our eCPR does not accurately record the timing of intermittent boluses given for maintenance of anaesthesia post-RSI. Therefore, we are unable to correlate maintenance boluses with the patient’s physiological observations at the time. Second, for same- and different-agent drug combinations, we are unable to assess the interval between induction and maintenance boluses, nor the temporal association between subsequent maintenance boluses. This temporal uncertainty is important, because the degree of pharmacological synergy between boluses of different drugs depends substantially on their relative timing. Moreover, published evidence quantifying synergistic or additive effects of drug combinations in bolus-based maintenance regimens is limited, making further analytical interpretation of combined-agent dosing strategies largely speculative. Hence, our examination of induction-maintenance dosing interactions was limited to correlative analysis, and we were prevented from any further detailed analysis of pharmacodynamic interactions between different-agent drug combinations that, through additive or synergistic effects, may produce adequate levels of sustained anaesthesia. Third, the study may have been underpowered to detect any relevant (confounding) associations between patient characteristics and maintenance dosing. Finally, to standardise intermittent boluses doses between cases, we took the pragmatic approach of dividing the summated bolus doses by weight and time to allow comparison. However, we acknowledge that the pharmaco-kinetics and -dynamics of intermittent boluses are different to an equivalent dose over the same time as a continuous infusion.

## Conclusion

This retrospective analysis demonstrated that prehospital anaesthesia maintenance by intermittent boluses may result in a potential risk of sub-therapeutic dosing for haemodynamically stable trauma patients with a GCS ≥ 9. Further, there was considerable inter-patient variability in dosing, unexplained by patient characteristics or transport duration. Restrictions in data recording limit the ability to assess potential pharmacodynamic interactions between agents, and associations between ongoing drug dosing and patient physiology. Continuous infusion protocols warrant investigation as a means to standardise maintenance dosing, off-load clinician cognitive burden, and reduce the risk of inadequate anaesthesia.

## Supplementary Information

Below is the link to the electronic supplementary material.

Supplementary Material 1

Supplementary Material 2

## Data Availability

The datasets used and/or analysed during the current study are available from the corresponding author on reasonable request.

## Abbreviations

KSS: Air Ambulance Charity Kent Surrey Sussex
ED: Emergency Department
GCS: Glasgow Coma Score
HEMS: Helicopter Emergency Medical Service
MTC: Major Trauma Centre
NIHR: National Institute for Healthcare Research
PHEA: Prehospital Emergency Anaesthesia
SOP: Standard Operating Procedure
PHEA: Prehospital Emergency Anaesthesia
RSI: Rapid Sequence Intubation

## References

[1] Lockey DJ, Healey B, Crewdson K, Chalk G, Weaver AE, Davies GE. Advanced airway management is necessary in prehospital trauma patients. Br J Anaesth. 2015;114:657–62. 10.1093/bja/aeu412. PMID: 25540067. 25540067 10.1093/bja/aeu412

[2] Fakhry SM, Scanlon JM, Robinson L, Askari R, Watenpaugh RL, Fata P, Hauda WE, Trask A. Prehospital rapid sequence intubation for head trauma: conditions for a successful program. J Trauma. 2006;60(5):997–1001. 10.1097/01.ta.0000217285.94057.5e. PMID: 16688061. 10.1097/01.ta.0000217285.94057.5e 16688061

[3] Bernard SA, Nguyen V, Cameron P, Masci K, Fitzgerald M, Cooper DJ, Walker T, Std BP, Myles P, Murray L, David, Taylor, Smith K, Patrick I, Edington J, Bacon A, Rosenfeld JV, Judson R. Prehospital rapid sequence intubation improves functional outcome for patients with severe traumatic brain injury: a randomized controlled trial. Ann Surg. 2010;252(6):959 – 65. 10.1097/SLA.0b013e3181efc15f. PMID: 21107105. 10.1097/SLA.0b013e3181efc15f 21107105

[4] Bossers SM, Mansvelder F, Loer SA, Boer C, Bloemers FW, Van Lieshout EMM, Den Hartog D, Hoogerwerf N, van der Naalt J, Absalom AR, Schwarte LA, Twisk JWR, Schober P, BRAIN-PROTECT Collaborators. Association between prehospital end-tidal carbon dioxide levels and mortality in patients with suspected severe traumatic brain injury. Intensive Care Med. 2023;49(5):491–504. 10.1007/s00134-023-07012-z. Epub 2023 Apr 19. PMID: 37074395; PMCID: PMC10205841. 37074395 10.1007/s00134-023-07012-z PMC10205841

[5] Lulla A, Lumba-Brown A, Totten AM, Maher PJ, Badjatia N, Bell R, Donayri CTJ, Fallat ME, Hawryluk GWJ, Goldberg SA, Hennes HMA, Ignell SP, Ghajar J, Krzyzaniak BP, Lerner EB, Nishijima D, Schleien C, Shackelford S, Swartz E, Wright DW, Zhang R, Jagoda A, Bobrow BJ. Prehospital Guidelines for the Management of Traumatic Brain Injury – 3rd Edition. Prehosp Emerg Care. 2023;27(5):507–38. Epub 2023 Apr 20. PMID: 37079803. 37079803 10.1080/10903127.2023.2187905

[6] Lyon RM, Perkins ZB, Chatterjee D, Lockey DJ, Russell MQ, Kent. Surrey & Sussex Air Ambulance Trust. Significant modification of traditional rapid sequence induction improves safety and effectiveness of pre-hospital trauma anaesthesia. Crit Care. 2015;19(1):134. 10.1186/s13054-015-0872-2. PMID: 25879683; PMCID: PMC4391675. 25879683 10.1186/s13054-015-0872-2 PMC4391675

[7] Lockey DJ, Crewdson K, Davies G, Jenkins B, Klein J, Laird C, Mahoney PF, Nolan J, Pountney A, Shinde S, Tighe S, Russell MQ, Price J, Wright C. AAGBI: Safer pre-hospital anaesthesia 2017: Association of Anaesthetists of Great Britain and Ireland. Anaesthesia. 2017;72(3):379–90. 10.1111/anae.13779. Epub 2017 Jan 3. PMID: 28045209; PMCID: PMC5324693. 28045209 10.1111/anae.13779 PMC5324693

[8] Hossfeld B, Bein B, Boettiger BW, Bohn A, Fischer M, Graesner JT, Hinkelbein J, Kill C, Lott C, Popp E, Roessler M, Schaumberg A, Wenzel V, Bernhard M. Recommended practice for out-of-hospital emergency anaesthesia in adults: Statement from the Out-of-Hospital Emergency Anaesthesia Working Group of the Emergency Medicine Research Group of the German Society of Anaesthesiology and Intensive Care. Eur J Anaesthesiol. 2016;33(12):881–897. 10.1097/EJA.0000000000000533. PMID: 27635954. 10.1097/EJA.0000000000000533 27635954

[9] Kottmann A, Krüger AJ, Sunde GA, Røislien J, Heltne JK, Carron PN, Lockey D, Sollid SJM. Establishing quality indicators for pre-hospital advanced airway management: a modified nominal group technique consensus process. Br J Anaesth. 2022;128(2):e143–50. Epub 2021 Oct 19. PMID: 34674835; PMCID: PMC8792832. 34674835 10.1016/j.bja.2021.08.031 PMC8792832

[10] Sheridan B, Perkins Z. Maintenance of prehospital anaesthesia in trauma patients: inconsistencies and variability in practice. BJA Open. 2025;13:100366. 10.1016/j.bjao.2024.100366. PMID: 39868410; PMCID: PMC11764628. 10.1016/j.bjao.2024.100366 PMC11764628 39868410

[11] Stocchetti N, Maas AI. Traumatic intracranial hypertension. N Engl J Med. 2014;370(22):2121-30. 10.1056/NEJMra1208708. PMID: 24869722. 10.1056/NEJMra1208708 24869722

[12] Di Battista AP, Rizoli SB, Lejnieks B, Min A, Shiu MY, Peng HT, Baker AJ, Hutchison MG, Churchill N, Inaba K, Nascimento BB, de Oliveira Manoel AL, Beckett A, Rhind SG. Sympathoadrenal Activation is Associated with Acute Traumatic Coagulopathy and Endotheliopathy in Isolated Brain Injury. Shock. 2016;46(3 Suppl 1):96–103. PMID: 27206278; PMCID: PMC4978599. 27206278 10.1097/SHK.0000000000000642 PMC4978599

[13] Johansson PI, Henriksen HH, Stensballe J, Gybel-Brask M, Cardenas JC, Baer LA, Cotton BA, Holcomb JB, Wade CE, Ostrowski SR. Traumatic Endotheliopathy: A Prospective Observational Study of 424 Severely Injured Patients. Ann Surg. 2017;265(3):597–603. PMID: 27144442; PMCID: PMC5300027. 27144442 10.1097/SLA.0000000000001751 PMC5300027

[14] de Kock JM, Buma C, Stassen W. A retrospective review of post-intubation sedation and analgesia practices in a South African private ambulance service. Afr J Emerg Med. 2022;12:467e72. 36415448 10.1016/j.afjem.2022.10.009 PMC9674874

[15] Cook TM, Andrade J, Bogod DG, Hitchman JM, Jonker WR, Lucas N, Mackay JH, Nimmo AF, O’Connor K, O’Sullivan EP, Paul RG, Palmer JH, Plaat F, Radcliffe JJ, Sury MR, Torevell HE, Wang M, Hainsworth J, Pandit JJ. Royal College of Anaesthetists and the Association of Anaesthetists of Great Britain and Ireland. The 5th National Audit Project (NAP5) on accidental awareness during general anaesthesia: patient experiences, human factors, sedation, consent and medicolegal issues. Anaesthesia. 2014;69(10):1102-16. 10.1111/anae.12827. PMID: 25204237. 10.1111/anae.12827 25204237

[16] Pappal RD, Roberts BW, Mohr NM, Ablordeppey E, Wessman BT, Drewry AM, Winkler W, Yan Y, Kollef MH, Avidan MS, Fuller BM. The ED-AWARENESS Study: A Prospective, Observational Cohort Study of Awareness With Paralysis in Mechanically Ventilated Patients Admitted From the Emergency Department. Ann Emerg Med. 2021;77(5):532–44. Epub 2021 Jan 21. PMID: 33485698; PMCID: PMC8166299. 33485698 10.1016/j.annemergmed.2020.10.012 PMC8166299

[17] Kim MC, Fricchione GL, Akeju O. Accidental awareness under general anaesthesia: Incidence, risk factors, and psychological management. BJA Educ. 2021;21(4):154–61. 10.1016/j.bjae.2020.12.001. Epub 2021 Jan 21. PMID: 33777414; PMCID: PMC7984969. 33777414 10.1016/j.bjae.2020.12.001 PMC7984969

[18] Billups K, Larrimore A, Li J, Werman H, Shirk MB. Impact of paralytic agent on postintubation sedation. Air Med J. 2019 Jan-Feb;38(1):39–44. 10.1016/j.amj.2018.09.006. Epub 2018 Oct 25. PMID: 30711084. 10.1016/j.amj.2018.09.006 30711084

[19] Bonomo JB, Butler AS, Lindsell CJ, Venkat A. Inadequate provision of postintubation anxiolysis and analgesia in the ED. Am J Emerg Med. 2008;26(4):469 – 72. 10.1016/j.ajem.2007.05.024. PMID: 18410818. 10.1016/j.ajem.2007.05.024 18410818

[20] Frakes MA, Lord WR. Sedative use in patients receiving neuromuscular blocking agents from a helicopter flight team. Air Med J. 2006 Jul-Aug;25(4):173-5. 10.1016/j.amj.2006.04.006. PMID: 16818168. 10.1016/j.amj.2006.04.006 16818168

[21] Crewdson K, Rehn M, Brohi K, Lockey DJ. Pre-hospital emergency anaesthesia in awake hypotensive trauma patients: beneficial or detrimental? Acta Anaesthesiol Scand. 2018;62(4):504–514. 10.1111/aas.13059. Epub 2018 Jan 7. PMID: 29315456. 10.1111/aas.13059 29315456

[22] ter Avest E, Ragavan D, Griggs J, Dias M, Mitchinson SA, Lyon R. Haemodynamic effects of a prehospital emergency anaesthesia protocol consisting of fentanyl, ketamine and rocuronium in patients with trauma: a retrospective analysis of data from a Helicopter Emergency Medical Service. BMJ Open. 2021;11(12):e056487. 10.1136/bmjopen-2021-056487. PMID: 34930748; PMCID: PMC8689168. 34930748 10.1136/bmjopen-2021-056487 PMC8689168

[23] Chesters A, Keefe N, Mauger J, Lockey D. Prehospital anaesthesia performed in a rural and suburban air ambulance service staffed by a physician and paramedic: a 16-month review of practice. Emerg Med J. 2014;31(1):65–8. 10.1136/emermed-2012-201846. Epub 2013 Jan 23. PMID: 23345316. 23345316 10.1136/emermed-2012-201846

[24] Hayes-Bradley C, Tarrant M. Rocuronium ≤ 1.5 mg/kg versus > 1.5 mg/kg and inadequate paralysis in prehospital and retrieval intubation: a retrospective study. Emerg Med Australas. 2022 Dec;34(6):892–7. 10.1111/1742-6723.14008. Epub 2022 Jun 1. PMID: 35649634. 10.1111/1742-6723.14008 35649634

[25] Hodkinson M, Poole K. Induction of pre-hospital emergency anaesthesia i-PHEA: a national survey of UK HEMS practice. BMC Emerg Med. 2023;23(1):126. 10.1186/s12873-023-00897-5. PMID: 37904097; PMCID: PMC10617087. 37904097 10.1186/s12873-023-00897-5 PMC10617087

[26] Griggs JE, Clarke S, Greenhalgh R, Watts AN, Barrett J, Houghton Budd S, Dias M, Hunter K, Lyon RM, Ter Avest E, Air Ambulance Charity Kent Surrey Sussex. Diagnostic accuracy of pre-hospital invasive arterial blood pressure monitoring for haemodynamic management in traumatic brain injury and spontaneous intracranial haemorrhage. Scand J Trauma Resusc Emerg Med. 2025;33(1):89. 10.1186/s13049-025-01393-4. PMID: 40380216; PMCID: PMC12082994. 40380216 10.1186/s13049-025-01393-4 PMC12082994

[27] Moy HP, Olvera D, Nayman BD, Pappal RD, Hayes JM, Mohr NM, Kollef MH, Palmer CM, Ablordeppey E, Faine B, Roberts BW, Fuller BM. The AIR-SED Study: A Multicenter Cohort Study of SEDation Practices, Deep Sedation, and Coma Among Mechanically Ventilated AIR Transport Patients. Crit Care Explor. 2021;3(12):e0597. 10.1097/CCE.0000000000000597. PMID: 34909700; PMCID: PMC8663813. 34909700 10.1097/CCE.0000000000000597 PMC8663813

[28] Sikorski RA, Koerner AK, Fouche-Weber LY, Galvagno SM. Choice of general anesthetics for trauma patients. Curr Anesthesiol Rep. 2014;4:225–32. 10.1007/s40140-014-0066-5.

[29] Zaphir JS, Murphy KA, MacQuarrie AJ, Stainer MJ. Understanding the Role of Cognitive Load in Paramedical Contexts: A Systematic Review. Prehosp Emerg Care. 2025;29(2):101–14. Epub 2024 Jul 30. PMID: 38922409. 38922409 10.1080/10903127.2024.2370491

