# Supplementary material for: Maintenance of prehospital anaesthesia using an intermittent bolus regime in blunt trauma patients with a high GCS and hemodynamic reserve: a retrospective cohort study
Source: Scand J Trauma Resusc Emerg Med. 2026 Jun 25;34:136. doi: 10.1186/s13049-026-01654-w (PMC13476940; doi:10.1186/s13049-026-01654-w)
Supplement: Supplementary file 1 [file 13049_2026_1654_MOESM1_ESM.docx]

**APPENDIX 1**

**Table 3. Comparative summative doses of maintenance drugs**

| **Maintenance Drug (Dose)** | **KSS Intermittent Bolus** | **AIR-SED ^a^** | | |
| --- | --- | --- | --- | --- |
|  | **Median Dose** | **Light Sedation Median Dose** | **Deep Sedation Median Dose** | **Total Median Dose** |
| **Fentanyl (mcg/kg/h)** | 0.64 | 0.99 | 1.11 | 1.02 |
| **Midazolam (mg/kg/h)** | 0.028 | 0.05 | 0.06 | 0.05 |
| **Ketamine (mg/kg/h)** | 0 | 2.05 | 2.49 | 2.29 |
| **Rocuronium(mg/kg/h)** | 0.43 | 0.84 | 0.89 | 0.81 |

**Legend Table 3.** AIR-SED, Ref [27]; ^a^ calculated from median cumulative dose (m(c)g) divided by median weight(kg) divided by duration of care (hr)

**Table 4. Comparison of individual bolus doses of maintenance drugs**

| **Maintenance Drug** | **KSS Intermittent Bolus** | | **South African Private EMS ^c^** | | | **German Out-of-Hospital Emergency Anaesthesia Working Group** | **MAP-RSI Survey** |
| --- | --- | --- | --- | --- | --- | --- | --- |
|  | **Most Common Bolus Dose** | **2nd Most Common Bolus Dose** | **Average 1st Bolus Dose** | **Average 2nd Bolus Dose** | **Average 3rd Bolus Dose** | **Recommended Bolus Dose** | **Reported Bolus Doses (weight-based)** |
| **Fentanyl (mcg)** | 100 | 50 | - | - | - | 150 | 10-40 (or 0.5mcg/kg) |
| **Midazolam (mg)** | 2 | 1 | 4.4 | 4 | 3.6 | 3-5 | 1-3 (or 0.5-<0.1mg/kg) |
| **Ketamine (mg)** | 20 | 30 | - | - | - | 20 ^d^ | 10-20 (or 0.1mg/kg) |
| **Rocuronium (mg)** | 40 | 30 | - | - | - | 20 | 50 (or 0.5-2.0mg/kg) |

**Legend Table 4.** South African Private EMS, Ref [14]; German Recommended Practice Statement, Ref [8]; MAP-RSI Survey, Ref [10]; ^c^ as single agent; ^d^ as esketamine
