# Supplementary material for: Maintenance of prehospital anaesthesia using an intermittent bolus regime in blunt trauma patients with a high GCS and hemodynamic reserve: a retrospective cohort study
Source: Scand J Trauma Resusc Emerg Med. 2026 Jun 25;34:136. doi: 10.1186/s13049-026-01654-w (PMC13476940; doi:10.1186/s13049-026-01654-w)
Supplement: Supplementary file 2 [file 13049_2026_1654_MOESM2_ESM.docx]

**Supplementary Table S1.** Complete correlation matrix for cross-drug maintenance dosing and induction dosing.

| **Maintenance Drug** | **Induction Drug** | **n** | **r** | **p-value** | **Bonferroni p** |
| --- | --- | --- | --- | --- | --- |
| Fentanyl | Fentanyl | 65 | -0.018 | 0.8896 | >0.999 |
| Fentanyl | Ketamine | 65 | 0.103 | 0.4125 | >0.999 |
| Fentanyl | Midazolam (pre) | 65 | -0.083 | 0.5102 | >0.999 |
| Fentanyl | Rocuronium | 65 | 0.059 | 0.6378 | >0.999 |
| Ketamine | Fentanyl | 29 | -0.470 | 0.0100* | 0.1606 |
| Ketamine | Ketamine | 29 | -0.243 | 0.2034 | >0.999 |
| Ketamine | Midazolam (pre) | 29 | -0.130 | 0.5008 | >0.999 |
| Ketamine | Rocuronium | 29 | -0.047 | 0.8069 | >0.999 |
| Midazolam | Fentanyl | 61 | 0.180 | 0.1657 | >0.999 |
| Midazolam | Ketamine | 61 | 0.134 | 0.3030 | >0.999 |
| Midazolam | Midazolam (pre) | 61 | 0.144 | 0.2695 | >0.999 |
| Midazolam | Rocuronium | 61 | 0.063 | 0.6278 | >0.999 |
| Rocuronium | Fentanyl | 70 | -0.002 | 0.9899 | >0.999 |
| Rocuronium | Ketamine | 70 | -0.162 | 0.1795 | >0.999 |
| Rocuronium | Midazolam (pre) | 70 | -0.013 | 0.9120 | >0.999 |
| Rocuronium | Rocuronium | 70 | -0.228 | 0.0579 | 0.9258 |

**Supplementary Table S1.** Pearson correlation coefficients (r) with uncorrected and Bonferroni-corrected p-values examining potential interactions between all induction drug doses (columns) and maintenance drug infusion rates (rows). This comprehensive analysis includes both same-drug correlations (e.g., fentanyl induction vs. fentanyl maintenance) and cross-drug correlations (e.g., ketamine induction vs. midazolam maintenance, reflecting the common clinical pathway where ketamine induces and midazolam maintains anaesthesia).Induction midazolam includes pre-induction dosing only (as midazolam is not used for induction in this cohort). Bonferroni correction applied for 16 multiple comparisons (adjusted α = 0.003125). Only patients receiving each respective maintenance drug are included; n varies due to zero maintenance doses and missing data. After correction, no induction-maintenance associations remained statistically significant, indicating maintenance dosing is independent of initial induction strategy. Asterisks denote uncorrected statistical significance: *p<0.05, **p<0.01, ***p<0.001.

**Supplementary Table S2.** Complete correlation matrix for maintenance dosing and pre-induction physiological parameters.

| **Drug** | **Physiological parameter** | **n** | **r** | **p-value** | **Bonferroni p** |
| --- | --- | --- | --- | --- | --- |
| Fentanyl | GCS | 65 | -0.106 | 0.3996 | >0.999 |
| Fentanyl | Heart Rate | 65 | 0.150 | 0.2335 | >0.999 |
| Fentanyl | Systolic BP | 65 | -0.057 | 0.6543 | >0.999 |
| Ketamine | GCS | 29 | 0.160 | 0.4062 | >0.999 |
| Ketamine | Heart Rate | 29 | 0.339 | 0.0717 | 0.8603 |
| Ketamine | Systolic BP | 29 | -0.121 | 0.5313 | >0.999 |
| Midazolam | GCS | 61 | 0.179 | 0.1682 | >0.999 |
| Midazolam | Heart Rate | 61 | -0.141 | 0.2767 | >0.999 |
| Midazolam | Systolic BP | 61 | 0.020 | 0.8796 | >0.999 |
| Rocuronium | GCS | 70 | -0.023 | 0.8491 | >0.999 |
| Rocuronium | Heart Rate | 70 | 0.002 | 0.9900 | >0.999 |
| Rocuronium | Systolic BP | 70 | -0.147 | 0.2237 | >0.999 |

**Legend Supplementary Table S2.** Pearson correlation coefficients (r) with uncorrected and Bonferroni-corrected p-values for associations between standardised per-kilogram-per-hour maintenance infusion rates and Glasgow Coma Scale (GCS), pre-induction heart rate, and pre-induction systolic blood pressure. Bonferroni correction applied for 12 multiple comparisons (adjusted α = 0.05/12 = 0.00417). These parameters complement the correlations shown in Figure 3. Only patients receiving each respective maintenance drug are included; n varies due to zero maintenance doses and missing data. GCS, Glasgow Coma Scale Score. BP, Blood pressure.
